# Early versus delayed laparoscopic cholecystectomy in acute cholecystitis: A prospective study

**DOI:** 10.6026/973206300220338

**Published:** 2026-01-31

**Authors:** Kaushlendra Singh Narwariya, Rakesh Shakya, Sandeep Sharma, Krishna Pratap Singh, Parul Nema

**Affiliations:** 1 Department of General Surgery, SRVS Medical College Shivpuri, Madhya Pradesh, India; 2 Department of General Surgery, Motilal Nehru Medical College Prayagraj, Uttar Pradesh, India; 3 Department of Pathology, SRVS Medical College Shivpuri, Madhya Pradesh, India

**Keywords:** Acute cholecystitis, laparoscopic cholecystectomy, surgical outcomes, operative time, hospital stay, postoperative complications

## Abstract

The optimal timing for laparoscopic cholecystectomy in patients with acute calculous cholecystitis remains unclear. Therefore, it is of interest to compare early versus delayed laparoscopic cholecystectomy in 246 patients with acute calculous cholecystitis. Early surgery performed within 72 hours showed significantly shorter operative time, reduced hospital stay and faster return to normal activity. Conversion to open surgery and postoperative complications were similar between both groups. Delayed surgery required longer operative duration and hospitalization despite comparable safety outcomes. Early laparoscopic cholecystectomy within 72 hours is more effective and efficient, with shorter operative time, reduced hospital stay and quicker recovery compared to delayed surgery.

## Background:

Acute calculous cholecystitis is a common emergency surgical condition and a frequent indication for cholecystectomy worldwide. The management of acute cholecystitis has evolved over the past two decades with laparoscopy becoming the preferred approach, but the optimal timing of cholecystectomy-whether during the index admission (early) or after initial conservative management (delayed)-remains a subject of ongoing investigation and debate. Recent guideline updates and systematic reviews generally support early operative management because of potential benefits such as shorter total hospital length of stay and overall resource use, while not showing a consistent increase in major morbidity compared with delayed surgery [1, 2]. Definitions of "early" versus "delayed" cholecystectomy vary between studies and guidelines, which complicate comparison between reports and the formulation of universal recommendations. Some guideline bodies and trials define early surgery as that performed within 72 hours of symptom onset or within the hospital admission, while others adopt a wider early window of up to 7-10 days from symptom onset; delayed or interval surgery is commonly performed after 6-8 weeks following clinical recovery. This heterogeneity in timing definitions is an important reason for persistent uncertainty regarding the optimal interval for surgery [1, 2-3]. Accumulating evidence from multicentre observational cohorts and meta-analyses indicates that early laparoscopic cholecystectomy is associated with shorter total hospital stay and faster return to normal activities without a clear increase in major complications or conversion rates compared with delayed intervention. Several recent large observational studies and systematic reviews have explored whether the "7-day barrier" (the notion that surgery beyond 7 days is inherently more hazardous) is clinically relevant; while operating time and intraoperative technical difficulty may increase with longer symptom duration, pooled data have not consistently demonstrated higher rates of major complications or conversion to open surgery when early surgery is performed beyond the first week [4]. Conversely, in clinical situations where early index-admission cholecystectomy is not feasible because of patient comorbidity, resource limitations, or initial conservative management, some series have suggested that interval cholecystectomy performed after a longer convalescent period may be associated with reduced operative time and comparable or even lower short-term morbidity; however, these findings are largely context- dependent and not universally reproducible. This diversity of findings underscores the need for further prospectively collected data comparing clinically relevant intraoperative and postoperative outcomes between early and delayed strategies using clear, pre-specified timing windows [5]. Therefore, it is of interest to compare intraoperative parameters, postoperative recovery and complication rates between early (index-admission) and delayed (interval) laparoscopic cholecystectomy for acute calculous cholecystitis, using predefined timing criteria and standardized outcome measures, in order to provide additional evidence to inform optimal timing in routine clinical practice.

## Methodology:

This prospective observational study was conducted at a tertiary care teaching hospital. Patients diagnosed with acute calculous cholecystitis, based on clinical presentation, laboratory findings and ultrasonographic evidence, were enrolled after obtaining written informed consent. The diagnosis of acute cholecystitis was established using the Tokyo Guidelines criteria [6], which included right upper quadrant pain, tenderness, fever, leukocytosis and ultrasonographic findings of gallbladder wall thickening or pericholecystic fluid. A total of 246 patients aged between 18 and 65 years, presenting within 72 hours of symptom onset, were included. They were divided into two groups of 123 each: Group A underwent early laparoscopic cholecystectomy (within 72 hours of admission), while Group B underwent delayed laparoscopic cholecystectomy after an initial course of conservative management and elective surgery performed 6-8 weeks later. Patients with empyema gallbladder, perforation, choledocholithiasis, severe comorbidities (ASA grade ≥ III), or previous upper abdominal surgeries were excluded from the study. All patients received standard preoperative evaluation, including hematological and biochemical investigations, liver function tests and abdominal ultrasonography. In the early surgery group, laparoscopic cholecystectomy was performed as soon as possible after stabilization. In the delayed group, initial conservative therapy included intravenous antibiotics, analgesics, hydration and bowel rest until clinical resolution of symptoms, followed by interval surgery. All surgeries were performed under general anesthesia by surgeons experienced in minimally invasive procedures, using a standard four-port technique. Intraoperative parameters such as duration of surgery, gallbladder wall thickness, adhesions, bile spillage, conversion to open cholecystectomy and intraoperative complications were documented. Postoperative outcomes including duration of hospital stay, wound infection, bile leak, postoperative pain (assessed using a visual analog scale) and time to resume normal activity were recorded. Patients were followed up at 1 week, 1 month and 3 months postoperatively for evaluation of late complications such as port-site infection or biliary stricture. All data were compiled and analyzed using SPSS software version 26.0. Continuous variables were expressed as mean ± standard deviation and categorical data as proportions or percentages. Comparison between groups was performed using the independent Student's t-test for continuous variables and the Chi-square test for categorical variables. A p-value of less than 0.05 was considered statistically significant.

## Results:

A total of 246 patients with acute calculous cholecystitis were enrolled, comprising 118 in the early laparoscopic cholecystectomy (ELC) group and 128 in the delayed laparoscopic cholecystectomy (DLC) group. Both groups were comparable with respect to baseline demographic and clinical characteristics (Table 1). The mean operative time was significantly shorter in the early surgery group (p<0.001). Although inflammatory adhesions were more frequent in ELC cases, the conversion rate to open surgery did not differ significantly (Table 2). Patients undergoing early surgery had significantly shorter hospital stays, earlier resumption of oral intake and faster recovery to normal activity. Postoperative pain scores were also marginally lower in the ELC group (Table 3). No statistically significant differences were observed between the two groups in postoperative complications. There were no cases of bile duct injury or mortality in either group (Table 4).

## Discussion:

The findings of our prospective study demonstrate that ELC in patients with acute calculous cholecystitis offers significant advantages in terms of reduced operative time, shorter hospital stay and faster recovery, without a detectable increase in conversion or complication rates when compared to DLC. These observations align with the growing body of evidence favouring early intervention in this clinical setting. Our operative time data show a statistically significant reduction in the ELC group versus the delayed group, suggesting that performing cholecystectomy during the index admission may mitigate progression of inflammation and adhesion formation that can complicate later procedures. This is consistent with findings from network meta-analysis work which reported that cholecystectomy performed within ≤72 hours reduced conversion rates compared to operations delayed for several weeks. That meta-analysis also noted that while delaying surgery may reduce operative time modestly, it may concurrently increase overall hospital stay and resource utilisation [7]. The shorter hospital length of stay and the faster return to normal activity in the ELC cohort underscore the economic and patient-centred benefits of early surgery. A multicenter study from two university hospitals reported that a longer preoperative waiting time was independently associated with a prolonged postoperative length of stay (median five vs nine days, p = 0.001) and emphasized the cost-saving potential of early definitive surgery [8]. These findings support our data showing that earlier surgical intervention not only improves clinical throughput but may also reduce healthcare burden.

Importantly, our complication rates-including wound infection, bile leak and conversions to open surgery-were comparable between groups and did not reach statistical significance. This concurs with recent prospective nonrandomized data which found ELC to result in fewer complications, fewer readmissions and shorter stays than DLC [9]. The absence of increased morbidity with early surgery in our study supports the safety of this approach when performed in appropriately selected patients. However, several caveats merit discussion. First, although early surgery may offer benefits, the technical difficulty in some cases can be higher because the inflammatory response is still active, potentially leading to more challenging dissection. Some investigators have proposed that operative complexity may increase with duration of symptoms prior to surgery, even if major complications do not [10]. Surgeons must therefore apply operative caution and ensure proper instrumentation, high laparoscopic skill and readiness for conversion. Second, the "delayed" window is not uniformly defined in surgical literature, with some series defining delayed cholecystectomy at 6-8 weeks and others at over 12 weeks. A retrospective study assessing interval surgery found no significant difference in conversion or complication rates between operations done at 1-6 weeks versus 6-12 weeks post-event, suggesting that interval cholecystectomy remains safe within that timeframe [11]. These data highlight that while early surgery is preferable, delayed surgery in certain contexts remains acceptable-but probably less optimal in terms of recovery metrics. In recent studies, Goel *et al.* (2025) [12] discussed the implications of biotechnological advancements in life sciences, shedding light on the evolving pharmaceutical research landscape and its application to disease management. Similarly, Meena *et al.* (2025) [13] explored the effectiveness of specific pharmaceutical interventions in the medical field, contributing valuable insights into treatment optimization and patient care.

Third, our study design, like other real‐world investigations, is constrained by potential selection bias: patients suitable for early surgery may have been healthier or less inflamed at baseline, although our baseline demographic and clinical matching aimed to limit this. Moreover, we performed the study in a single tertiary centre, which may limit generalisability to settings with limited laparoscopic resources or less experienced surgeons. Future multicentre randomized trials would further strengthen evidence regarding timing. In terms of clinical implications, our results suggest that, in institutions with competent laparoscopic capability and appropriate patient selection, early laparoscopic cholecystectomy should be the preferred strategy in acute cholecystitis. It offers quicker recovery, reduced hospital utilisation and no detectable increase in adverse surgical outcomes. For patients who, however, present late, or in whom early surgery is contraindicated due to comorbidities or unavailability of OR capacity, delayed laparoscopic cholecystectomy remains a safe alternative-yet the delay should not be unnecessarily prolonged beyond the timeframe where inflammation is active and adhesions are forming. While recognizing the limitations of non-randomisation and single-centre context, our study adds to the accumulating evidence favouring early laparoscopic cholecystectomy in acute calculous cholecystitis. It supports implementation of institutional protocols aimed at reducing time-to-surgery from admission where feasible, with the aim of improving patient-centred outcomes and optimising resource use.

## Conclusion:

Early laparoscopic cholecystectomy in patients with acute calculous cholecystitis is a safe and effective intervention, providing significant advantages over delayed surgery, including shorter operative time, reduced hospital stay, faster return to normal activity and comparable complication rates. Thus, we show early definitive surgical management as the preferred strategy in appropriately selected patients, while delayed cholecystectomy remains a safe alternative when early surgery is not feasible. Adoption of early intervention protocols in clinical practice can improve patient-centered outcomes and optimize healthcare resource utilization.

## Figures and Tables

**Table 1:** Baseline demographic and clinical characteristics

**Parameter**	**Early LC (n = 118)**	**Delayed LC (n = 128)**	***p-value***
Mean age (years)	43.6 ± 10.8	45.1 ± 11.2	0.28
Male : Female ratio	01:01.3	01:01.4	0.72
Mean BMI (kg/m^2^)	25.7 ± 2.8	26.1 ± 3.0	0.41
Duration of symptoms before admission (hours)	36.4 ± 10.2	37.8 ± 11.5	0.39
WBC count (x10^9^/L)	12.3 ± 2.2	12.6 ± 2.4	0.31
Associated comorbidities (Diabetes/Hypertension)	26 (22.0%)	30 (23.4%)	0.78

**Table 2:** Intraoperative findings

**Parameter**	**Early LC (n = 118)**	**Delayed LC (n = 128)**	***p-value***
Mean operative time (minutes)	58.2 ± 12.5	67.6 ± 14.1	<0.001
Difficult dissection (Calot's triangle inflammation/adhesions)	38 (32.2%)	24 (18.8%)	0.014
Bile spillage	22 (18.6%)	16 (12.5%)	0.17
Conversion to open cholecystectomy	6 (5.1%)	10 (7.8%)	0.42
Intraoperative bleeding >100 mL	5 (4.2%)	8 (6.3%)	0.45

**Table 3:** Postoperative outcomes

**Outcome**	**Early LC (n = 118)**	**Delayed LC (n = 128)**	***p-value***
Mean hospital stay (days)	3.4 ± 1.1	6.7 ± 1.5	<0.001
Postoperative pain score (VAS, 0-10) at 24h	3.2 ± 0.9	3.6 ± 1.0	0.006
Time to oral intake (hours)	8.5 ± 2.4	10.7 ± 3.1	<0.001
Return to normal activity (days)	5.1 ± 1.2	7.8 ± 1.6	<0.001

**Table 4:** Postoperative complications

**Complication**	**Early LC (n = 118)**	**Delayed LC (n = 128)**	***p-value***
Wound infection	5 (4.2%)	9 (7.0%)	0.33
Bile leak	2 (1.7%)	3 (2.3%)	0.74
Postoperative fever	6 (5.1%)	10 (7.8%)	0.42
Residual collection (USG detected)	1 (0.8%)	2 (1.6%)	0.6
Readmission within 30 days	2 (1.7%)	3 (2.3%)	0.74
